# Characterization of Targeted Phenolic Compounds in Globe Artichoke Heads and Waste from Vegetatively and “Seed”-Propagated Genotypes

**DOI:** 10.3390/plants12132579

**Published:** 2023-07-07

**Authors:** Anna Bonasia, Giulia Conversa, Corrado Lazzizera, Antonio Elia

**Affiliations:** Department of Agriculture, Food, Natural Resources and Engineering (DAFNE), University of Foggia, 71100 Foggia, Italy; anna.bonasia@unifg.it (A.B.); corrado.lazzizera@unifg.it (C.L.); antonio.elia@unifg.it (A.E.)

**Keywords:** *Cynara cardunculus* L. subsp. *scolymus*, Brindisino, Violetto di Foggia, Tempo, Opal, Madrigal, polyphenols, chlorogenic acid, cynarin, luteolin, apigenin

## Abstract

In the globe artichoke, both the edible portion and the waste biomass are recognized as valuable sources of bioactive compounds. For this study, heads with 30 cm–long floral stems including two to three leaves were harvested from five genotypes, which included two traditional vegetative-propagated varietal types (“Brindisino” and “Violetto di Foggia”) and three “seed”-propagated hybrids (“Tempo”, “Opal”, and “Madrigal”). The study aimed to determine the total and individual polyphenolic concentrations (measured spectrophotometrically and using HPLC) and antioxidant activity (AA) in different artichoke parts, namely the “hearts” (H), head waste (HW), stem waste (SW), and leaf waste (LW). “Brindisino” SW exhibited the highest accumulation of luteolin (26,317 mg kg^−1^ F.W.), while “Tempo” H displayed the highest cynarin content (190 mg kg^−1^ F.W.). “Tempo” HW and H showed the highest levels of apigenin (640 mg kg^−1^ F.W.), and the greatest source of chlorogenic acid was found in the HW of “Opal” and the H of “Brindisino” (4300 mg kg^−1^ F.W.). The hybrids generally exhibited lower total polyphenolic concentrations than the traditional genotypes, particularly evident in the LW. The SW demonstrated the highest concentration of total polyphenols (18,000 mg kg^−1^ F.W.), followed by the edible H and non-edible HW (12,000 mg kg^−1^ F.W.), while the LW exhibited the lowest concentration (2000 mg kg^−1^ F.W.). Interestingly, the AA did not precisely align with the total polyphenolic concentration, showing slight variations between the examined parts and genotypes.

## 1. Introduction

Italy is the main producer of globe artichoke (*Cynara cardunculus* L. subsp. *scolymus* (L.) Hayek), Hegi) in the European frame, and the Puglia region (South of Italy) is the most prominent Italian producer. In this region, the cultivation of globe artichoke is mainly based on vegetative-propagated varietal types, represented mainly by the violet-green–colored “Violetto di Foggia” (a “Violet du Provence” type) (Figure S1a), cultivated in Foggia province, and the green-violet–colored “Brindisino” (a “Catanese” type) (Figure S1b), cultivated in Brindisi province and protected with the European recognition of “Protected Geographical Indication”.

Along with the traditional vegetative-propagated varietal types, some hybrids that can be “seed”-propagated have also been cultivated for about 20 years in Italy [1] and the Puglia region [2,3], representing 30% of the whole cultivation area at the time of writing (personnel communication). These hybrids are usually cultivated as an annual crop instead of traditional multi-year cycles [4]. The main “seed”-propagated hybrids that have spread in recent years are classified according to plant earliness, head biometry, and end-use. In the Puglia region, the hybrid Madrigal (Figure S1c) (quite late), with a light-green head, is mainly used by the processing industry, while the “Opal” (early) (Figure S1d) and “Tempo” (late) hybrids (Figure S1e), both with a purple head and green shades, are grown for both the fresh market and industry.

Globe artichoke heads are sent worldwide for fresh markets or industry as canned, frozen, and fresh-cut products [5]. The edible part of the head is represented by the “heart”, including the enlarged receptacle, budding flowers, and inner and tender bracts. Thus, from the head (approx. 40%; Bonasia et al. [6]) but also plant cultivation (approx. 85%; Francavilla et al. [7]) of globe artichoke, a huge amount of waste is continuously generated from industrial processing (non-edible parts of the head from the various food-use preparations) and from the fields (solid plant waste often left in the field) [8].

Both vegetative and reproductive parts of globe artichoke contain several phytochemicals (inulin, minerals, vitamin C, inositols, terpenoids, such as sesquiterpene lactones, and polyphenolic substances [9,10]. The healthy properties of artichoke extracts are mostly related to polyphenolic compounds [8,11], represented by phenolic acids—mainly chlorogenic acid, cynarin, and caffeic acid—and by flavonoids—specifically, apigenin and luteolin derivatives [12].

In a circular economy paradigm, waste materials from the industrial preparation of “hearts” and globe artichoke cultivation should be valorized as a resource of these bioactive compounds.

It is well known that the concentration and the profile of polyphenolic compounds of globe artichoke (and antioxidant activities) are influenced by biotic (genotype, plant tissue/organs, pathogens) and abiotic (environments, agronomic practices, harvesting time, etc.) conditions [9,13,14]. 

In the literature, the effects of biotic factors, genotype, and plant parts (edible and non-edible) on the polyphenolic concentration and profile have been extensively studied in traditional varieties [15,16,17,18,19]. However, published data on the newest genotypes are limited to the plant parts. Thus, the edible and non-edible parts (wastes) from traditional and “seed”-propagated genotypes of globe artichoke have been investigated in the present study, with special emphasis on the quantitative and qualitative profile of phenolic compounds and antioxidant activity.

## 2. Results and Discussion

The study presents a qualitative and quantitative evaluation of total polyphenol content using a spectrophotometric assay and the profile of targeted polyphenolic compounds through HPLC analysis. Four parts (one edible and three non-edible) of five different artichoke genotypes, including two vegetatively propagated and three “seed”-propagated types, were examined. Additionally, the study analyzed the dry matter content and total antioxidant activity.

Table 1 displays the results for the dry matter (DM) concentration of both edible and non-edible parts of the artichoke. Among the genotypes in the H part, “Madrigal”, “Violetto di Foggia”, and “Opal” exhibited the lowest average DM concentration (120.6 g kg^−1^ F.W.), as shown in Table 1. Conversely, “Tempo” had the highest DM concentration, consistent with previous research by Bonasia et al. [6].

In the SW part, “Tempo” displayed the highest DM concentration, while “Violetto di Foggia” showed the lowest, as indicated in Table 1. For the HW part, the genotype did not affect the DM concentration, averaging 150.4 g kg^−1^ F.W. (Table 1). Pandino et al. [18] focused on the physical and qualitative characteristics of the “Tempo” hybrid grown in Sicily, reporting a higher DM concentration (190 g kg^−1^ F.W.) in the outermost bracts compared to our study (155 g kg^−1^ F.W.; Table 1). However, this difference may be attributed to variations in the processing methods. The cited work considered only the 15 outermost bracts as waste, while our study included at least 30 bracts.

In the LW part, “Brindisino” exhibited the highest DM concentration, while “Tempo” and “Madrigal” displayed the lowest (Table 1). On average, the vegetatively propagated genotypes showed higher DM concentrations than the “seed”-propagated ones (Table 1). Notably, the DM of leaves cultivated in the Puglia region had a relatively higher average (126 g kg^−1^ F.W.) (Table 1) compared to the genotypes cultivated in Tunisia (97 g kg^−1^ F.W.) [20].

The study utilized spectrophotometric determination to assess the concentration of total phenols (TP), as presented in Table 2. Among the genotypes in the H part, “Violetto di Foggia” exhibited the highest TP concentration, followed by “Brindisino”. Conversely, the H of all “seed”-propagated hybrids displayed the lowest TP concentration, averaging 1984 mg a.g.e. kg^−1^ F.W. These compounds contribute to the nutritional value of the hearts of vegetatively propagated genotypes. On the other hand, the lower phenolic concentration in “seed”-propagated hybrids suggests an advantage for industrial and fresh-cut processing, reducing browning during storage, aligning with previous studies [5,21].

In detail, “Violetto di Foggia” demonstrated the highest TP concentration in the SW and LW parts (Table 2), while “Tempo” had the highest TP concentration in the HW part (Table 2). Overall, the vegetatively propagated genotypes exhibited higher TP concentrations compared to the “seed”-propagated ones in all examined parts (Table 2).

**Figure 1:**
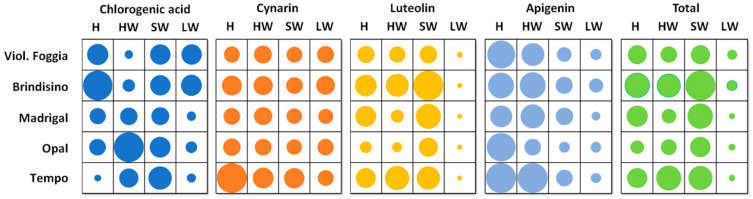
Relative abundance of the single phenol and their total as affected by genotype and plant part. H, “hearts”; HW, head waste; SW, stem waste; LW, leaf waste.

Additionally, the phenolic profile, obtained through HPLC analysis, varied significantly between genotypes and parts, as presented in Table 3 and Figure 1.

Within the H part, “Brindisino” displayed the highest total polyphenol (TP) content (135 g kg^−1^ D.W.), primarily consisting of luteolin (74%) and chlorogenic acid (23%) (Table 3 and Figure 1). Conversely, “Opal” had the lowest TP content (47 g kg^−1^ D.W.), with luteolin as the major constituent (67%) (Table 3 and Figure 1). In “Tempo” H, cynarin (1.1 g kg^−1^ D.W.) and apigenin (4 g kg^−1^ D.W.) were present in higher concentrations, while the chlorogenic acid content was lower (1.4 g kg^−1^ D.W.) (Table 3 and Figure 1).

For the HW part, “Tempo” and “Brindisino” stood out with the highest TP concentrations (116 g kg^−1^ D.W.), primarily attributed to the abundant luteolin content (101 g kg^−1^ D.W., representing 80% of the total) (Table 3 and Figure 1). “Tempo” also exhibited the highest cynarin (0.5 g kg^−1^ D.W.) and apigenin (4 g kg^−1^ D.W.) concentrations in the HW part (Table 3 and Figure 1). In the study by Pandino et al. [22], “Tempo”-HW displayed significantly lower total and individual polyphenol levels (1.4 g kg^−1^ D.W. vs. 116 g kg^−1^ D.W.) and a different profile, with no detection of caffeoylquinic acids, such as cynarin and chlorogenic acid. Among the flavonoids, apigenin derivatives were the most abundant (96% of the total). Notably, “Opal”-HW exhibited the highest concentration of chlorogenic acid (35 g kg^−1^ D.W.; 58% of the total) (Table 3 and Figure 1).

In the SW part, “Brindisino” and “Violetto di Foggia” had the highest and lowest total phenol concentrations, respectively, mainly because of variations in luteolin levels (193 and 80 g kg^−1^ D.W., respectively; Table 3 and Figure 1). “Tempo” showed the highest cynarin concentration (0.5 g kg^−1^ D.W.) (Table 3), while “Opal” had the lowest apigenin concentration in the SW part (0.7 g kg^−1^ D.W.; Table 3).

In terms of TP accumulation, there was a noticeable difference between the “seed”- and vegetative-propagated genotypes in the LW part. The “seed”-propagated genotypes accumulated lower TP levels compared to the vegetative-propagated ones (1400 vs. 3100 mg kg^−1^ F.W.; 126 vs. 154 g kg^−1^ D.W.) primarily because of lower amounts of all identified compounds, except for luteolin, which remained unaffected by genotype (Table 3; Figure 1). The TP contents in the LW of the three “seed”-propagated hybrids and specific varieties, such as “Opal” and “Madrigal” (Table 3), were lower than those found in leaves of seven hybrids (3930 mg kg^−1^ F.W. on average), particularly “Opal” (4700 mg kg^−1^ F.W.) and “Madrigal” (4070 mg kg^−1^ F.W.) grown hydroponically [23]. The advanced horticultural system, characterized by high density, protected culture, and a short cycle, likely contributed to higher antioxidant concentrations compared to our leaves grown in traditional and open-air systems. However, the polyphenolic profile in our LW (Table 3) was consistent with the findings of Rocchetti et al. [23], where flavonoids were the predominant class of phenolics, while caffeoylquinic acids were less abundant. 

In general, the “seed”-propagated genotypes exhibited lower total polyphenol concentrations compared to the vegetative-propagated ones for each examined part (Table 3), consistent with the results from spectrophotometric analyses (Table 2). According to HPLC data, the TP content was lower by 11% in the HW, 14% in the SW, 26% in the H, and 56% in the LW (Table 3). These differences can be attributed to lower amounts of the most abundant components, such as luteolin among flavonoids, especially in the HW (29% reduction) and the SW (16% reduction), as well as chlorogenic acid among the caffeoylquinic acids in the H (71% reduction) and the LW (76% reduction) (Table 3).

Luteolin, a flavonoid, was the predominant component, accounting for 83% of the total, except for the LW part, where luteolin represented 38% of the total, while caffeoylquinic and chlorogenic acids accounted for 55% of the total (Table 3 and Figure 1). In contrast, previous studies by Pandino et al. [24] and Pandino et al. [22] found that, regardless of genotypes, cultivation year, and other agronomic experimental treatments in globe artichoke leaves, luteolin derivatives were the main compounds, followed by caffeoylquinic acids and apigenin derivatives. The content trend of luteolin > caffeoylquinic acids > apigenin observed in the H part (Table 3) was not consistent with the capitula of three traditional Italian varieties grown in Sicily (without distinction in parts), which showed apigenin (98.7%) > caffeoylquinic acids (0.9%) > luteolin (0.4% of the total) [25]. 

The quantitative and qualitative profile of polyphenols is widely described in the literature, indicating that it depends on various factors, including genotype, environmental conditions, and agronomic practices [23,26,27].

Flavonoids, such as luteolin and apigenin flavones, are not commonly found in food plants [28]. However, globe artichoke, particularly waste from plants like “Brindisino” SW and waste from heads like “Tempo” HW, can serve as significant sources of these bioactive compounds. HW and H, in general, exhibited the highest levels of apigenin flavones (Table 3; Figure 1). Flavones have demonstrated potential biological effects both in vitro and in vivo [29].

Among the two detected caffeoylquinic acids, chlorogenic acid was the most abundant (Table 3; Figure 1). Regardless of genotype, the SW (2000 mg kg^−1^ F.W.; 15 g kg^−1^ D.W. on average) and the LW (1100 mg kg^−1^ F.W.; 9 g kg^−1^ D.W. on average) contained distinctly higher levels of chlorogenic acid compared to recent studies characterizing polyphenols in globe artichoke biomass from artichoke cultivations (in Ingallina et al. [10], leaves were 7.5 g kg^−1^ D.W., and stalks were 2.3 g kg^−1^ D.W.). Specifically, for Madrigal LW, the concentration of chlorogenic acid was found to be higher (Table 3; 10 mg kg^−1^ D.W.) than in the leaf biomass of the same genotype (3.9 g kg^−1^ D.W.), as reported by Francavilla et al. [7]. This difference can likely be attributed to the age and position of the organs examined in our study, where only a portion of floral stems with two-three leaves was analyzed, whereas the cited studies [7,10] considered bulk residual biomass from artichoke crops containing leaves and stems at different stages. Furthermore, El Senousy et al. [30] reported that the top-positioned leaf of the artichoke plant is a better source of polyphenolic compounds compared to the basal leaves.

Cynarin, a compound belonging to (di)caffeoylquinic acids, had the lowest concentration in each organ, ranging from 0.3 to 2.8% of the total, depending on the part (0.4–0.6 g kg^−1^ D.W.) (Table 3; Figure 1). The cynarin concentration varied between 190 mg kg^−1^ F.W. (1.1 g kg^−1^ D.W.) in “Tempo” H and 50 mg kg^−1^ F.W. (0.4 g kg^−1^ D.W.) in SW and LW of the “seed”-propagated varieties (Table 3). However, these values were considerably higher than the levels found in “Opal” and “Madrigal” hybrids grown in the Metapontino Plain (Matera province, South Italy), which ranged from 1.1 mg kg^−1^ F.W. in external bracts to 15.0 mg kg^−1^ F.W. in inner bracts [27].

Despite its relatively low content in globe artichoke, cynarin has been found to significantly enhance the nutraceutical effects of this vegetable (Gezer, 2017). Cynarin has shown potential health effects, including choleretic and cholesterol-lowering properties, hepatoprotective effects, anti-atherosclerotic activity, anti-HIV activity, antioxidative properties, anti-diabetic effects, anti-carcinogenic properties, and immune modulation [31,32].

Regardless of the genotype examined, the SW exhibited the highest concentration of total and individual polyphenols, with an average of 18,000 mg kg^−1^ F.W. (140 g kg^−1^ D.W.), followed by the edible H and the non-edible HW, with an average of 12,000 mg kg^−1^ F.W. (90 g kg^−1^ D.W.). The LW had the lowest concentration, averaging 2000 mg kg^−1^ F.W. (16 g kg^−1^ D.W.) (Table 3). Therefore, the observed trend in our study was SW > H = HW > LW.

Our results are consistent with a previous study by Della Gatta and Patruno (1973), reported in Di Venere et al. [33], which examined the distribution of total polyphenols in artichoke heads and floral stems. They found that the portion of the stem proximate to the head displayed the highest concentration of polyphenols compared to other parts of the head, such as bracts and receptacles. Lombardo et al. [19], as reviewed by Pandino et al. [34], also reported that floral stems had the highest capacity for polyphenol accumulation when measured using a colorimetric method, compared to the edible and inedible parts of the head.

Regarding HW, Pandino et al. [18] highlighted the high capacity for polyphenol accumulation in the outermost bracts, which was higher than that observed in the edible parts (receptacle and inner bracts). Interestingly, Pandino et al. [18] reported a much lower level of polyphenols in the outermost bracts (4.4 g kg^−1^ D.W. on average) compared to our findings in head waste (Table 3; 11,700 mg kg^−1^ F.W.; 85 g kg^−1^ D.W. on average). In contrast, Frattianni et al. [15] conducted a detailed examination of polyphenol distribution in different parts of the head and young leaves of several genotypes. They found that the inner bracts exhibited the highest polyphenol accumulation, followed by the receptacle and intermediate bracts, while the outermost bracts and leaves had the lowest levels.

Antioxidant activity (AA) plays a crucial role in evaluating the quality of vegetables as antioxidant molecules contribute to plant growth and development under stressful conditions, as well as promoting health benefits in the human diet [35]. AA, determined using the β-carotene/linoleate assay and presented in Table 4, was significantly influenced by the genotype in each examined plant part. “Tempo” exhibited the highest AA in LW while displaying the lowest AA in the H, HW, and SW (along with “Opal” and “Violetto di Foggia”). On the other hand, “Violetto di Foggia” demonstrated the lowest AA in the LW and the highest in the HW and H (along with “Madrigal” and “Opal”) (Table 4).

On average, the AA values of LW were slightly lower compared to the literature findings based on genotype and plant part. In our study, the AA of LW ranged from 31% to 55% (with a mean of 48%) (Table 4), while values close to 59% were reported for leaves of a genotype cultivated in Tunisia using the same assay [20].

Further exploring the “Brindisino” genotype, the AA values aligned with results obtained from the same variety grown in the Puglia region by Lattanzio et al. [36]. In that study, AA reached 58% in HW and 66% in H.

The observed AA did not entirely correlate with TP data determined through spectrophotometric and HPLC methods (Table 2, Table 3 and Table 4). Some discrepancies were found between TP concentration and AA, such as low TP concentration coupled with high AA. It is plausible to hypothesize that other antioxidative compounds, including vitamin C, not analyzed in our study, could have contributed to the observed increase in AA. This phenomenon was evident in the H of “Opal” and “Madrigal” (seed-propagated varieties) and in the SW of “Madrigal”. Dabbaou et al. [37] and Petropoulos et al. [38] confirmed, based on their experiments with artichoke leaves and tissues, that the free scavenging activity of artichokes is not solely attributed to phenolic compound concentration. The lack of a strong correlation between TP concentration and AA could also be attributed to the limitations of the AA determination method employed. According to several authors [39,40], the antioxidant potency is dependent on the extraction and assay methodologies. Nickavar and Esbati [41] stated that the radical scavenging activities of the adopted methodology are influenced by the substrate’s polarity, specifically its hydro-/lipo-philicity. Therefore, we suggest that this method may be more suitable for analyzing the lipophilic component of the matrix.

### Principal Component Analysis 

**Figure 2:**
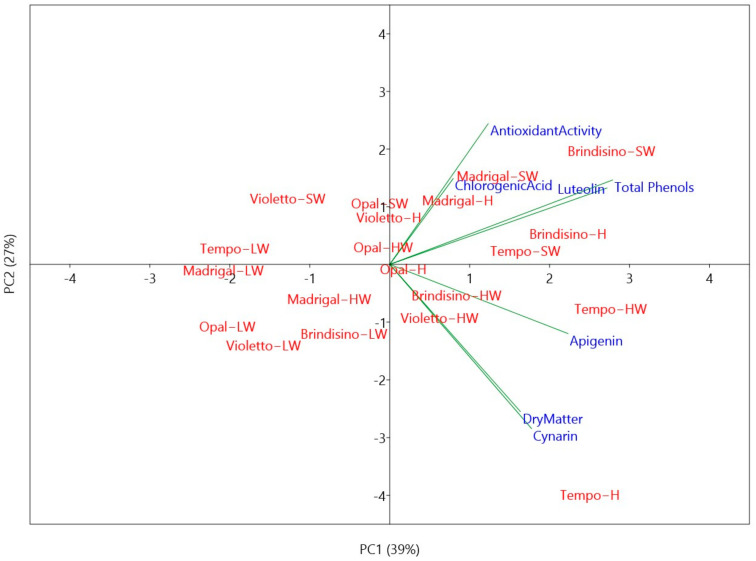
Principal component analysis bi-plot (PC1 vs. PC2) showing quality traits of globe artichoke as a function of organs (H, “hearts”; HW, head waste; SW, stem waste; LW, leaf waste) and genotypes (by vegetative propagation: “Brindisino”, “Violetto di Foggia” named Violetto; by “seed” propagation: “Madrigal”, “Tempo”, “Opal”). According to previous correlation studies between polyphenolic concentration data from HPLC and spectrophotometric analysis, only data from HPLC analysis were considered.

To comprehensively analyze the phenolic changes influenced by genotype and different plant parts (edible “hearts”—H, non-edible waste, head waste—HW, leaf waste—LW, stem waste—SW), a principal component analysis (PCA) was performed on the entire dataset. The first two principal components (PCs) had eigenvalues greater than 1 and collectively accounted for 66% of the total variance, with PC1 explaining 39% and PC2 explaining 27%. Since PC1 and PC2 captured significant variance, they were selected for interpretation (Figure 2).

PC1 exhibited a strong positive correlation (>0.5) with total polyphenolic concentration, luteolin, and apigenin. On the other hand, PC2 showed a positive correlation with antioxidant activity and chlorogenic acid while displaying a negative correlation with cynarin and dry matter content. The PCA scores effectively separated and categorized the treatments into distinct groups, facilitating the interpretation of results based on all the examined parameters.

The upper right quadrant of the PCA plot represents genotypes and organs characterized by high antioxidant activity (particularly in “Brindisino” and “Madrigal” SW), as well as elevated total phenolic and luteolin contents (especially in “Brindisino” H). The cluster in the lower right quadrant represents HW of “Tempo” and “Brindisino”, which exhibited high levels of apigenin. Additionally, “Tempo” H was characterized by high cynarin and dry matter content, as confirmed by the ANOVA results (Table 1 and Table 3). Furthermore, the upper and lower left quadrants correspond to LW samples from all examined varieties, displaying the lowest values for all the considered traits.

## 3. Materials and Methods

### 3.1. Plant Material

Artichoke heads, with floral stems (30 cm long, bearing 2–3 leaves), were harvested from 5 genotypes: 2 traditional vegetative-propagated varietal types (“Brindisino” and “Violetto di Foggia”) and 3 “seed”-propagated hybrids (“Tempo”, “Opal”, “Madrigal”) (Nunhems Netherlands BV, Haelen, The Netherlands) (Figure 1). “Brindisino” (a “Catanese” type) was collected from commercial fields in Brindisi province (40°37′57.821″ N 17°56′30.343″ E), while the other 4 genotypes were from Foggia province (41°27′43.914″ N 15°32′40.669″ E), Puglia region (South Italy). 

Four parts were considered for each genotype: the “hearts” (H); the head waste (HW); the stem waste (SW), represented by 30 cm–long floral stems; the leaf waste (LW), represented by the 2–3 leaves borne by floral stems. 

The HW included the 30 outermost and inedible leathery bracts + the apex of the head (cut across at 70% of head height); additionally, all bracts showing a cutting force (a digital pressure tester, model 53205, TR; Turoni & C. s.n.c., Forlì, Italy) higher than a threshold value (35 N), as described in Bonasia et al. [6], were also included in the HW.

### 3.2. Measurements

The four parts (H, HW, SW, LW) considered for each genotype were analyzed for dry matter (DM) concentration, phenolic concentration and profile, and antioxidant activity.

Fresh samples of each part (F.W.) were dried at 65 °C in a thermo-ventilated oven until a constant weight was achieved to obtain the dry weight (D.W.). The DM concentration was determined as the D.W./F.W. ratio.

Fresh plant material was sliced into small pieces, treated with liquid nitrogen, and subsequently lyophilized (model Lio5P; CinquePascal s.r.l., Trezzano, Milano, Italy). 

The lyophilized sample (1 g) was used for extraction with 2 × 20 mL of water:methanol (20:80 *v*:*v*) solution; the extract was then centrifuged at 14,000 rpm for 15 min at 4 °C (Beckman Coulter AllegraTM 25; Fullerton, CA, USA). The supernatant was extracted twice, recovered, and filtered through a filter paper. The filtrate was considered as artichoke extract and kept in the dark at –20 °C until being tested for the following assays. 

Total phenolic content was determined on methanolic extract according to the method of Singleton and Rossi [42]. The methanolic extract (100 μL) was mixed with 0.5 mL of Folin–Ciocalteu reagent and allowed to stand at room temperature for 5 min; then, 1.0 mL of sodium bicarbonate solution Na_2_CO_3_ (20%) was added to the mixture. After 45 min at 30 °C, absorbance was read at 750 nm. Results were expressed as gallic acid equivalents using the calibration curve.

The profiles of phenolic compounds were determined using HPLC-DAD analysis according to Baiano et al. [43]: HPLC binary system (Agilent, model G1311A; Santa Clara, CA, USA) equipped with a 7725 Rheodyne injector, a 20-µL sample loop, a diode array detector (DAD) (Agilent, model G1315Bm), and a ChemStation integrator (Agilent) for data acquisition. The stationary phase was a Nova-Pack C18 analytical column (150 × 3.9 mm) with a particle size of 4 µm (Waters, Milford, MA, USA). 

The mobile phases for chromatographic analysis were (A) water:acetic acid (98:2, *v*:*v*) and (B) methanol:acetonitrile (1:1, *v*:*v*) at a constant flow rate of 1 mL/min. The gradient program of solvent was as follows: 0 to 30 min 100% A; 30 to 45 min 70% A; 45 to 55 min 50% A; 55 to 65 min 40% A; 65 to 75 min 0% A.

HPLC was calibrated using commercial standards provided by Sigma–Aldrich (St. Louis, MO, USA): chlorogenic acid (≥96% purity), 1,3-dicaffeoylquinic acid (≥95% purity) (cynarin), apigenin (95% purity), and luteolin (≥95% purity). The identification of phenolic components was carried out by comparing the peak retention times with those obtained by injection of pure standards. 

The antioxidant activity of artichoke methanolic extract was assayed based on the linoleic acid/β-carotene bleaching assay [44]. The mixture of β-carotene and linoleic acid was prepared by dissolving 0.5 mg of β-carotene, 25 µL of linoleic acid, and 200 µL of Tween 40 in 1 mL of chloroform solvent. The chloroform was evaporated in a rotator evaporator at 40 °C, and 100 mL of dH_2_O was added; then, the mixture was stirred. Aliquots of 2.5 mL of β-carotene/linoleic acid emulsion obtained were transferred to test tubes; then, the emulsion was incubated for 2 h at 50 °C, and the absorbance of each sample was measured at 470 nm by spectrophotometer. The β-carotene bleaching is detectable through the absorbance decrease, which is greater when the antioxidant content is low. The antioxidant activity was expressed as a percentage using the following formula: AA = [(initial absorbance Sample) − (final absorbance Sample)]/[(initial absorbance Control − final absorbance Control)].

### 3.3. Statistical Analysis

The statistical analysis was performed with the Statistical Analysis System software using the General Linear Model (GLM Proc of the SAS Software; SAS 9.1; SAS Institute, Cary, NC, USA). The comparison between the means was performed by calculating the least significant difference (LSD) (*p* = 0.05). For visual analysis of data, principal component analysis (PCA) was performed using the PAST3 Software (http://folk.uio.no/ohammer/past (accessed on 3 May 2023).) on mean standardized ((x-mean)/standard deviation) data. The data matrix considered all genotypes and portions with relative replications.

## 4. Conclusions

Both genotype and plant part had a significant impact on phenolic content. The “seed”-propagated genotypes, despite having lower total polyphenolic levels compared to the vegetative-propagated ones, exhibited notable concentrations of individual phenolic compounds. In particular, “Tempo” demonstrated distinctive characteristics in the non-edible parts, such as HW, with high levels of apigenin (640 mg kg^−1^ F.W.; 4 g kg^−1^ D.W.), and in the edible part (H), it stood out for cynarin content (190 mg kg^−1^ F.W.; 1.1 g kg^−1^ D.W.). “Madrigal” displayed remarkable levels of chlorogenic acid and antioxidant activity in both the non-edible SW and the edible H.

Among the vegetative-propagated genotypes, “Brindisino” exhibited a rich profile of phenolic compounds in both the edible and non-edible parts. Overall, SW and HW, which are typically considered industrial or domestic waste, demonstrated their potential as interesting sources of natural antioxidants.

The floral stems, which are approximately 30 cm long and were considered in this study, are usually discarded during industrial or domestic preparation of the artichoke H. However, they possess significant amounts of phenolic compounds, with the floral stems (16,000 mg kg^−1^ F.W.; 121 g kg^−1^ D.W.) and HW (10,000 mg kg^−1^ F.W.; 69 g kg^−1^ D.W.) containing appreciable luteolin content. Moreover, the floral stems can be considered as an edible product, particularly in “seed”-propagated varieties, because of their wide and tender peduncle diameter. This suggests that they can serve as a significant source of nutraceutical compounds for consumers (artichoke products usually include the peduncle) while also providing an alternative source of revenue for producers and processors.

In addition to the typical utilization of artichoke heads in the fresh market or industry, exploring specific applications for the non-edible parts of the tested genotypes can promote the recovery of functional and bioactive compounds with known relevance in the food, feed, therapeutic, cosmetic, and nutraceutical sectors.

## Figures and Tables

**Table 1:** The concentration of dry matter in different parts of five globe artichoke genotypes.

Genotype	Dry Matter Concentration (g kg^−1^ F.W.)			
	H ^(1)^	HW	SW	LW
Violetto di Foggia	113.3 ± 4.7 b ^(2)^	157.7 ± 1.8 a	105.3 ± 7.4 b	137.7 ± 9.3 ab
Brindisino	136.3 ± 0.8 ab	152.0 ± 15 a	125.7 ± 6.6 ab	158.3 ± 0.8 a
Madrigal	123.7 ± 1.4 b	144.3 ± 0.8 a	117.3 ± 0.3 ab	106.3 ± 1.7 c
Opal	125.0 ± 2.5 b	142.7 ± 3.2 a	117.7 ± 0.8 ab	128.0 ± 9.5 bc
Tempo	165.7 ± 21 a	154.7 ± 1.3 a	144.7 ± 18 a	102.3 ± 11 c
Significance ^(3)^	*	ns	*	*

^(1)^ H, “hearts”; HW, head waste; SW, stem waste; LW, leaf waste. ^(2)^ Within each part, the same lowercase letters in the column indicate that the mean values (±SE) are not significantly different (*p* = 0.05). ^(3)^ Significance: * for *p* ≤ 0.05; ns, not significant.

**Table 2:** The concentration of total phenols in different parts of five globe artichoke genotypes.

Genotype	Total Phenol Concentration (mg a.g.e. kg^−1^ F.W.) ^(1)^			
	H ^(2)^	HW	SW	LW
Violetto di Foggia	6435 ± 189 a ^(3)^	7671 ± 172 b	6163 ± 413 a	6447 ± 163 a
Brindisino	3823 ± 111 b	6842 ± 228 c	3606 ± 170 b	5050 ± 409 b
Madrigal	1879 ± 113 c	1743 ± 51 e	1459 ± 36 d	1411 ± 81 d
Opal	2228 ± 140 c	2816 ± 148 d	2846 ± 52 c	3567 ± 274 c
Tempo	1845 ± 62 c	8361 ± 272 a	3819 ± 126 b	5707 ± 365 ab
Significance ^(4)^	***	***	***	***

^(1)^ a.g.e., acid gallic equivalent. ^(2)^ H, “hearts”; HW, head waste; SW, stem waste; LW, leaf waste. ^(3)^ Within each part, the same lowercase letters in the column indicate that the mean values (±SE) are not significantly different (*p* = 0.05). ^(4)^ Significance: *** for *p* ≤ 0.001.

**Table 3:** Concentration of phenolic compounds in different parts of five globe artichoke genotypes.

	Concentration of Phenols (mg kg^−1^ F.W.)				
Genotype	Chlorogenic Acid	Cynarin	Luteolin	Apigenin	Total
	H ^(1)^				
Violetto di Foggia	2208 ± 838 b ^(2)^	54.6 ± 1.6 b	8495 ± 81 bc	553.1 ± 8.5 ab	11310 ± 894 b
Brindisino	4248 ± 117 a	83.7 ± 21 b	13578 ± 1466 a	474.0 ± 77 bc	18383 ± 1615 a
Madrigal	1266 ± 139 bc	56.3 ± 2.9 b	12514 ± 1956 ab	340.1 ± 29 c	14177 ± 2037 ab
Opal	1306 ± 31 bc	61.8 ± 4480 b	3880 ± 394 c	578.2 ± 39 ab	5826 ± 461 c
Tempo	232 ± 18 c	190.3 ± 32 a	11673 ± 1669 ab	639.3 ± 21 a	12735 ± 1706 b
Significance ^(3)^	***	**	**	*	**
	HW ^(1)^				
Violetto di Foggia	347 ± 54 b ^(2)^	66.6 ± 1.7 bc	7641 ± 913 b	373.4 ± 18 c	8429 ± 946 b
Brindisino	773 ± 102 b	78.1 ± 6.2 b	15321 ± 1953 a	483.8 ± 59 b	16656 ± 1986 a
Madrigal	1426 ± 75 b	67.7 ± 3.9 bc	4728 ± 724 bc	418.9 ± 22 bc	6640 ± 655 b
Opal	4371 ± 883 a	59.6 ± 0.8 c	2976 ± 292 c	199.0 ± 15 d	7605 ± 660 b
Tempo	1742 ± 309 b	90.4 ± 2.1 a	16717 ± 3273 a	647.1 ± 22 a	19197 ± 3469 a
Significance ^(3)^	**	**	**	***	***
	SW ^(1)^				
Violetto di Foggia	1976 ± 398 a ^(2)^	53.1 ± 4.1 b	9116 ± 332 c	148.4 ± 25 ab	11293 ± 740 d
Brindisino	1997 ± 826 a	58.2 ± 3.4 b	26317 ± 714 a	204.8 ± 16 ab	28577 ± 1471 a
Madrigal	1545 ± 435 a	50.0 ± 1.6 b	18245 ± 1530 b	217.9 ± 39 a	20058 ± 1111 b
Opal	2086 ± 359 a	49.7 ± 0.7 b	10826 ± 1601 c	85.2 ± 6.4 b	13046 ± 1948 cd
Tempo	2596 ± 323 a	86.9 ± 10.5 a	15724 ± 2009 b	195.1 ± 49 ab	18601 ± 2244 bc
Significance ^(3)^	ns	**	***	*	***
	LW ^(1)^				
Violetto di Foggia	2004 ± 589 a ^(2)^	60.2 ± 4.6 ab	987.7 ± 191 a	92.0 ± 19 b	3144 ± 623 a
Brindisino	2138 ± 381 a	72.5 ± 2.5 a	659.3 ± 171 a	141.6 ± 9.9 a	3011 ± 558 a
Madrigal	434 ± 48 b	48.1 ± 0.9 b	703.0 ± 151 a	53.9 ± 4.2 b	1240 ± 140 b
Opal	655 ± 106 b	55.9 ± 4.0 b	706.0 ± 111 a	79.8 ± 14 b	1497 ± 225 b
Tempo	433 ± 13 b	51.2 ± 8.6 b	783.3 ± 267 a	75.2 ± 16 b	1343 ± 293 b
Significance ^(3)^	**	*	ns	*	*

^(1)^ H, “hearts”; HW, head waste; SW, stem waste; LW, leaf waste. ^(2)^ Within each part, the same lowercase letters in the column indicate that the mean values (±SE) are not significantly different (*p* = 0.05). ^(3)^ Significance: ***, **, and * for *p* ≤ 0.001, 0.01, and ≤0.05, respectively; ns, not significant.

**Table 4:** Antioxidant activity in different parts of five globe artichoke genotypes.

Genotype	Antioxidant Activity (%)			
	H ^(1)^	HW	SW	LW
Violetto di Foggia	75.5 ± 19 a ^(2)^	66.9 ± 16 a	63.4 ± 20 b	31.0 ± 22 d
Brindisino	65.8 ± 22 b	61.8 ± 17 ab	71.6 ± 13 a	55.8 ± 21 b
Madrigal	74.7 ± 19 a	64.9 ± 10 ab	70.5 ± 23 a	54.3 ± 19 b
Opal	76.4 ± 15 a	64.1 ± 19 ab	63.6 ± 25 b	39.6 ± 24 c
Tempo	41.7 ± 32 c	60.6 ± 12 b	63.4 ± 19 b	64.0 ± 15 a
Significance ^(3)^	**	*	**	**

^(1)^ H, “hearts”; HW, head waste; SW, stem waste; LW, leaf waste. ^(2)^ Within each part, the same lowercase letters in the column indicate that the mean values (±SE) are not significantly different (*p* = 0.05). ^(3)^ Significance: * and ** for *p* ≤ 0.05 and 0.01, respectively.

## Data Availability

The data supporting the findings of this study are available from the corresponding authors upon request.

## Supplementary Materials

The following supporting information can be downloaded at https://www.mdpi.com/article/10.3390/plants12132579/s1, Figure S1: Genotype.
