# Supplementary material for: Characterization of Targeted Phenolic Compounds in Globe Artichoke Heads and Waste from Vegetatively and “Seed”-Propagated Genotypes
Source: Plants (Basel). 2023 Jul 7;12(13):2579. doi: 10.3390/plants12132579 (PMC10347044; doi:10.3390/plants12132579)
Supplement: Supplementary file 1 [file plants-12-02579-s001.zip › plants-2471574-supplementary.pdf]

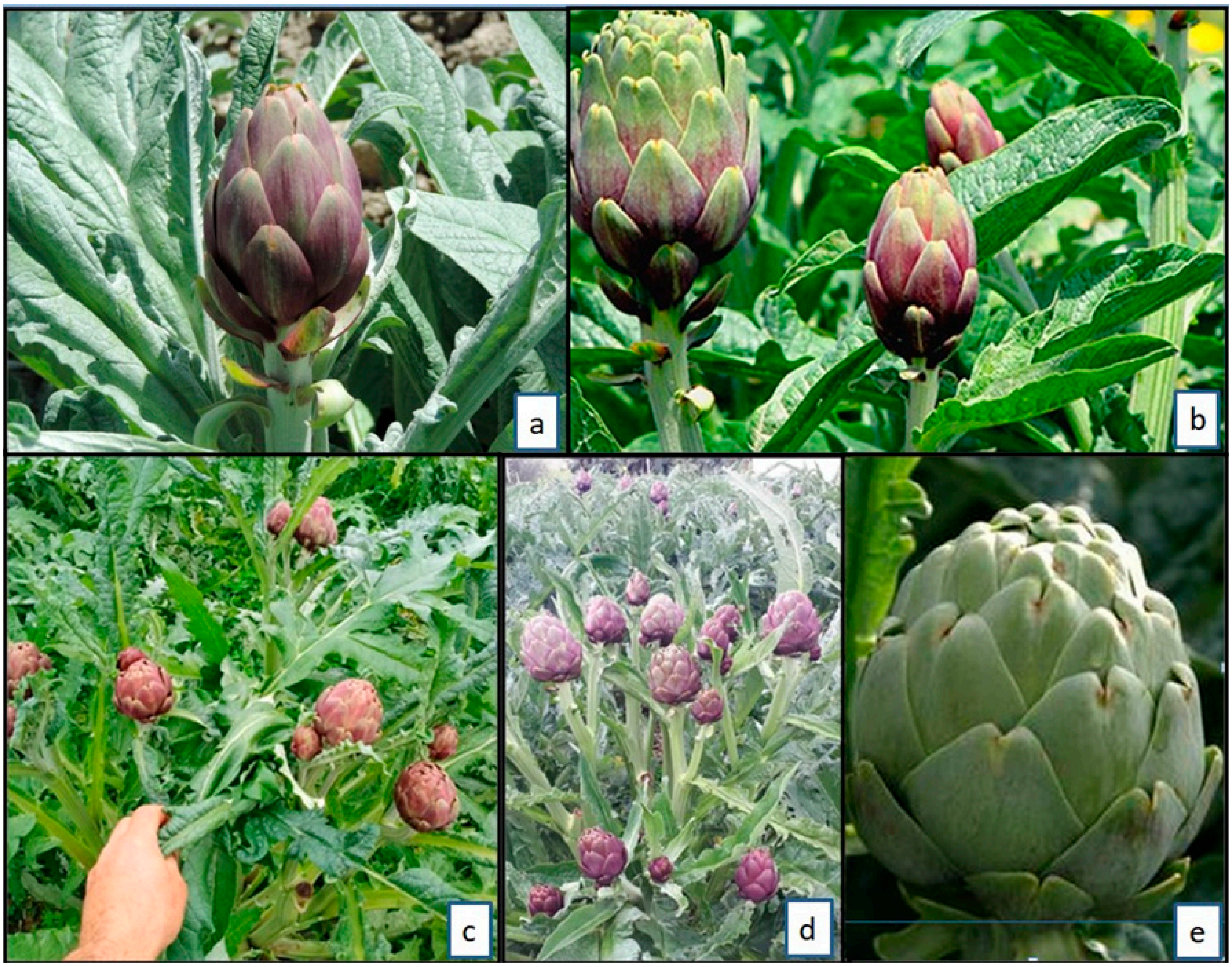

Figure S1 – Vegetatively-propagated varietal types: a) ‘Violetto di Foggia’; b) ‘Brindisino’; “Seed”-propagated varieties: c) ‘Tempo’; d) ‘Opal’; e) ‘Madrigal’.
